# Development of Clinical Neuroradiology: Sustained Growth and Increasing International Recognition

**DOI:** 10.1007/s00062-026-01714-9

**Published:** 2026-08-18

**Authors:** Martin Bendszus

**Affiliations:** Neurologische Klinik, Abteilung für Neuroradiologie, Universität Heidelberg, Heidelberg, Germany

The past three years have marked a period of remarkable growth and transformation for *Clinical Neuroradiology*. It is therefore my great pleasure to share some of the journal’s recent achievements and to express my sincere gratitude to everyone who has contributed to this success.

The most recent Journal Impact Factor has increased from 2.6 to 3.2, which further strengthens the position of *Clinical Neuroradiology* as one of the leading dedicated neuroradiology journals worldwide. With an Impact Factor of 3.5, the *American Journal of Neuroradiology (AJNR)* remains only marginally ahead, illustrating how substantially the gap between the two journals has narrowed over recent years.

The journal has also significantly improved its standing within the Journal Citation Reports. In the category Clinical Neurology, *Clinical Neuroradiology* is currently ranked 117 of 296 journals, corresponding to Quartile 2, representing an improvement of 21 positions compared with 2023. In the category *Radiology, Nuclear Medicine & Medical Imaging*, the journal is now ranked 64 of 217 journals, likewise in Quartile 2, after advancing by 14 positions during the same period. In this category, only approximately 10 ranking positions now separate *Clinical Neuroradiology* from Quartile 1, highlighting the remarkable progress that has been achieved.

Perhaps the most striking development has been the extraordinary increase in manuscript submissions. Within only three years, annual submissions have approximately tripled to an estimated 1200 manuscripts to be received in 2026. An increasing proportion of these submissions originates from internationally leading research institutions, further enhancing the scientific quality, visibility, and international reputation of the journal.

Despite this unprecedented increase in submissions, the editorial office has maintained an efficient and highly competitive editorial process. In 2025, the current median time to first decision was 6 days. Maintaining these turnaround times despite continuously increasing submission numbers has required an exceptional commitment from everyone involved in the editorial process.

On behalf of the entire editorial team, I would like to express my sincere appreciation to our Associate Editors and the members of the Editorial Board. Their scientific expertise, editorial judgment, and tireless dedication have been indispensable in managing the journal’s rapid development while maintaining the highest standards of scientific quality.

My deepest gratitude goes to our reviewers. High-quality peer review remains the foundation of scientific publishing. The thoughtful, constructive, and timely evaluations provided by our reviewers not only safeguard the scientific integrity of the journal but also provide invaluable guidance to authors in improving their work. Their voluntary commitment is one of the principal reasons for the continued success of *Clinical Neuroradiology*.

Despite these encouraging developments, one major challenge remains. Identifying suitable reviewers for increasingly specialized manuscripts continues to be one of the most important limiting factors in the editorial process and remains a significant contributor to prolonged manuscript handling times.

We therefore warmly invite colleagues with recognized expertise in all fields of neuroradiology to join our reviewer community. Researchers interested in serving as reviewers are encouraged to contact the journal at cnr@neuroradiologie.de, indicating their areas of scientific expertise. Expanding our international reviewer pool will further improve both the efficiency and the quality of the peer-review process while supporting the continued growth of the journal.

Finally, on behalf of the entire Editorial Team, I would like to thank all authors, reviewers, Associate Editors, Editorial Board members, readers, and the publishing staff at Springer Nature for their continued trust, enthusiasm, and support. The success of *Clinical Neuroradiology* is truly a shared achievement, and we look forward to continuing this exciting journey together.

Martin Bendszus, MD

Editor-in-Chief

Clinical Neuroradiology

## Data Availability

All data supporting the findings of this work are included in the article.

